# Neural Heterogeneity Underlying Behavioral Equivalence: A Dynamic Neuro‐Decoding Study of Cognitive and Affective Empathy in Relation to Autism‐Like Traits

**DOI:** 10.1002/brb3.71236

**Published:** 2026-01-28

**Authors:** Lingyu Zhao, Yixin Jiang, Ziwei Chen, Yongning Song

**Affiliations:** ^1^ School of Psychology and Cognitive Science East China Normal University Shanghai China; ^2^ Center For Faculty Development Southwest University Chongqing China; ^3^ Shanghai Key Laboratory of Brain Functional Genomics, Key Laboratory of Brain Functional Genomics (Ministry of Education), School of Psychology and Cognitive Science East China Normal University Shanghai China

**Keywords:** affective empathy, autism‐like traits, cognitive empathy, neural representation

## Abstract

**Purpose:**

Although empathy processing varies significantly between individuals with high and low autism‐like traits (ALT), the underlying neural mechanisms remain poorly understood. This study integrated behavioral measures with electroencephalography (EEG) analyses to investigate neural processing differences between high‐ and low‐ALT groups during cognitive and affective empathy tasks.

**Method:**

We assessed cognitive empathy (judging depicted emotions) and affective empathy (rating personal emotional resonance) in 40 participants (21 high‐ALT and 19 low‐ALT) using the Multifaceted Empathy Test while concurrently recording EEG data. Our analytical approach combined traditional behavioral analysis with multivariate pattern analysis and representational similarity analysis.

**Results:**

Behaviorally, the groups showed no significant performance differences. However, their neural mechanisms diverged. During cognitive empathy, the high‐ALT group exhibited inefficient early sensory processing and delayed, discontinuous neural encoding of emotion categories, suggesting reliance on late‐stage compensatory processing. For affective empathy, while early automatic neural resonance was intact, the high‐ALT group exhibited atypically sustained reliance on low‐level sensory features and failed to form stable, integrated neural representations of emotion, unlike the low‐ALT group.

**Conclusion:**

These findings reveal a pattern of “behavioral equivalence, neural heterogeneity.” High‐ALT individuals appear to employ distinct, compensatory neural strategies to achieve typical behavioral outcomes in empathy. This highlights the value of advanced neuro‐decoding in uncovering latent processing differences underlying the ALT spectrum.

## Graphical abstract

Although individuals with high and low autism‐like traits (ALT) perform identically on empathy tasks, neuro‐decoding reveals distinct underlying neural strategies. High‐ALT individuals employ late compensatory processing for cognitive empathy and fail to form integrated neural representations for affective empathy, demonstrating a pattern of behavioral equivalence despite neural heterogeneity.

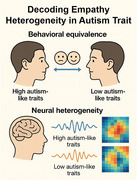

## 1 Introduction

Autism spectrum disorder (ASD) is a neurodevelopmental disorder characterized by persistent social communication impairments and restricted, repetitive behavioral patterns (American Psychiatric Association 2013). This core social dysfunction is widely attributed to deficits in social cognition, particularly difficulties in accurately interpreting the mental states of others (e.g., thoughts, intentions, emotions), which are foundational to social interaction (Dziobek et al. 2008; Georgiou et al. 2019; B.‐L. Le et al. 2024). These social‐cognitive difficulties are considered a primary cause of empathy deficits, which in turn impede prosocial behaviors (Dvash and Shamay‐Tsoory 2014; Oliveros et al. 2023; Vilas et al. 2021). Empathy, a critical capacity for maintaining interpersonal relationships, is typically divided into cognitive empathy (CE) and affective empathy (AE; aan het Rot and Hogenelst 2014; de Waal and Preston 2017; Decety 2011; Shamay‐Tsoory and Lamm 2018). CE is the ability to infer and understand the mental states of others (de Jonge et al. 2025; Liu et al. 2023; Rueda et al. 2015). AE involves sharing or resonating with the emotions of others, resulting in a vicarious emotional experience (B.‐L. Le et al. 2024; Liu et al. 2023; Rueda et al. 2015).

Research on empathy in ASD has revealed a dissociated pattern of deficits. A relatively consistent finding is that compared with typically developing (TD) individuals, the ASD cohort demonstrates impaired empathy (de Jonge et al. 2025). Specifically, numerous questionnaire‐based studies confirm that ASD individuals exhibit significant deficits in CE, whereas their AE often remains relatively intact compared to TD peers (Deschamps et al. 2014; Oliveros et al. 2023; Pouw et al. 2013; Shirayama et al. 2022; Simantov and Uzefovsky 2024; Ziermans et al. 2019). Experimental studies using tasks such as the multifaceted empathy test further support this finding, showing that ASD individuals perform significantly worse than TD peers in the CE task but not in the AE task (Dziobek et al. 2008). This dissociation extends to emotional valence: CE appears impaired for both positive and negative emotions, whereas AE deficits are observed primarily for negative emotions (Liu et al. 2023). However, this “CE‐impaired, AE‐intact” model is not absolute. For example, some studies report intact CE but impaired AE (Shalev et al. 2022), while others find impairments in both dimensions (Georgiou et al. 2019; Kimmig et al. 2024). These inconsistencies may stem from two key factors: the high heterogeneity within ASD individuals (Bora et al. 2017; de Jonge et al. 2025) and methodological differences across studies. For instance, self‐report questionnaires assessing stable “trait empathy” may be more sensitive to associations with autistic traits than behavioral tasks measuring immediate “state empathy” (Donaldson et al. 2022), but they are also more susceptible to confounds such as social desirability bias (Mul et al. 2018). Indeed, some studies report discrepancies where trait questionnaires detect group differences that are absent in behavioral tasks (McKenzie et al. 2022).

Autism‐like traits (ALTs) are subclinical manifestations of ASD that are continuously distributed throughout the general population (aan het Rot and Hogenelst 2014; Fletcher‐Watson and Bird 2020; Ruzich et al. 2015). Studies of high‐ALT individuals have similarly revealed an empathy profile similar to that of clinical ASD populations. Using measures such as the autism‐spectrum quotient (AQ) or the social responsiveness scale, studies consistently demonstrate a significant negative correlation between ALT and CE, whereas correlations with AE fail to reach significance (Brazil et al. 2024; Fletcher‐Watson and Bird 2020; B.‐L. Le et al. 2024). Similarly, high‐ALT individuals perform significantly worse on CE than low‐ALT individuals, with no group differences on AE (Grove et al. 2014). These findings support a “CE‐impaired, AE‐intact” pattern in subclinical populations. Nevertheless, these findings are not entirely consistent. For example, some studies report that AQ scores are negatively correlated with both CE and AE (Donaldson et al. 2022; McKenzie et al. 2022; Vilas et al. 2021) or find that group differences exist for both dimensions (B. Le et al. 2025). Conversely, other studies have found no association between ALTs and either empathy dimension (Mul et al. 2018; Oliver et al. 2016). These discrepancies may be explained by the measurement tools used, as stable trait questionnaires are more likely to reveal associations with ALTs than are behavioral tasks that measure empathy as a transient state (Donaldson et al. 2022; Massullo et al. 2020). Therefore, investigating the impact of ALTs on empathy requires careful consideration of the assessment methods and the specific dimensions they measure.

Several theoretical models attempt to explain the observed dissociation between CE and AE in both clinical ASD and high‐ALT populations. The classic theory of mind (ToM) deficit hypothesis posits that the core difficulty in ASD is an impaired ability to infer the mental states of others, which directly causes CE deficits (Baron‐Cohen 2009; Baron‐Cohen and Wheelwright 2004; Decety and Michalska 2010; Dvash and Shamay‐Tsoory 2014). In contrast, the Double Empathy Problem proposes that empathy difficulties reflect a reciprocal breakdown in understanding between ASD and TD individuals, stemming from differences in emotional processing and empathic style (Milton 2012; W. Zhang et al. 2023). This hypothesis further suggests that existing empathy assessments, which are normed on TD individuals, may be inherently biased (W. Zhang et al. 2023). The empathy imbalance model posits that CE and AE are two distinct yet interacting dimensions (Rueda et al. 2015; Shamay‐Tsoory et al. 2009; Smith 2009). In this model, the CE‐AE imbalance is considered not a deficit but a unique mode of empathic experience (Rueda et al. 2015; Shalev and Uzefovsky 2020). For instance, an AE‐dominant pattern has been linked to social difficulties (Ruzich et al. 2015; Shalev et al. 2023; Shalev and Uzefovsky 2020) and lower systemizing tendencies in ASD (Shalev and Uzefovsky 2020). Individuals with this pattern may experience intense vicarious emotions via AE but lack the CE to understand or regulate them, leading to emotional overload and social withdrawal (Smith 2009). Conversely, an opposite pattern could explain cases of AE impairment with relatively preserved CE (Shalev et al. 2022).

CE and AE are supported by dissociable, yet interacting neural networks. CE primarily relies on prefrontal cortical regions involved in mentalizing and executive functions, such as the dorsolateral prefrontal cortex and ventromedial prefrontal cortex, whereas AE engages limbic and paralimbic structures such as the amygdala and anterior insula, which are crucial for vicarious emotional experience (Almeida et al. 2024; de Waal and Preston 2017; Shirayama et al. 2022). Although functionally separable, two dimensions influence each other during social interaction (Rueda et al. 2015). ASD and high‐ALT individuals often show atypical activity in key nodes of these social‐brain networks. For example, amygdala dysfunction is frequently reported, including both hypoactivation (Arioli et al. 2021) and hyper‐responsivity to specific stimuli (e.g., faces, especially eyes; Fletcher‐Watson and Bird 2020). This hyper‐responsivity may trigger adaptive behaviors, such as gaze aversion, hindering further social information processing. Similarly, activity in the fusiform gyrus, a region critical for face processing, is often reduced (Zalla and Sperduti 2013). Moreover, activation in the anterior insular cortex may also be reduced (Y.‐T. Fan et al. 2014). Crucially, the neural circuits implicated in empathy substantially overlap with those involved in interoception, that is, the processing of one's own internal bodily and emotional states, which is a core deficit in alexithymia (B.‐L. Le et al. 2024; Yang et al. 2022).

Despite research unveiling potential mechanisms of empathy in individuals with ASD and high‐ALT, several key challenges remain. First, contradictory findings highlight methodological limitations such as self‐report bias (Dziobek et al. 2008; Mul et al. 2018), the low ecological validity of static tasks (McKenzie et al. 2022; Schnitzler and Fuchs 2024; Shamay‐Tsoory and Lamm 2018), and poor comparability across paradigms (Almeida et al. 2024). Second, similar behavioral outcomes may mask distinct underlying neural mechanisms. This issue underscores the need to supplement behavioral measures with analyses of underlying neural representations (Decety and Michalska 2010; Mazza et al. 2014; Oliver et al. 2016; W. Zhang et al. 2023). Therefore, a multimodal investigation of the ALT–empathy relationship can help validate the ALT continuum (aan het Rot and Hogenelst 2014; Brazil et al. 2024; Fletcher‐Watson and Bird 2020) and provide evidence for the neural basis of social‐cognitive diversity (Oliver et al. 2016).

To address these issues, the present study combines behavioral measures with multivariate pattern analysis (MVPA) and representational similarity analysis (RSA) to investigate the neural processing of CE and AE in high‐ and low‐ALT individuals. While traditional univariate EEG analyses (e.g., event‐related potentials, ERPs) focus on the magnitude and latency of neural responses at specific electrodes, they may overlook fine‐grained, distributed patterns of information. In contrast, MVPA leverages the full spatial‐temporal patterns of brain activity to decode the “quality” and stability of neural representations. Furthermore, RSA extends this by quantifying the “representational geometry,” enabling a direct test of whether neural activity is driven by high‐level emotion categories or low‐level sensory features. This approach is particularly crucial for distinguishing whether behavioral equivalence stems from identical neural processes or from compensatory strategies involving different information‐processing pathways. To ensure comparability with extant literature, we also examined classic ERP components (e.g., N100, N250) that index early emotional resonance and cognitive evaluation (Almeida et al. 2024; Y.‐T. Fan et al. 2014; Groen et al. 2013). These univariate results serve as a complementary baseline and are detailed in the Supporting Information.

We formulated three specific hypotheses. First, behaviorally, we hypothesized that the high‐ALT group would show deficits in CE but not in AE relative to the low‐ALT group, consistent with the “CE‐impaired, AE‐intact” model. Second, using MVPA, we aimed to compare the temporal stability of emotion decoding between the groups, hypothesizing that high‐ALT individuals would exhibit less stable neural encoding. Finally, using RSA, we tested whether neural representations in the high‐ALT group would be disproportionately driven by low‐level stimulus features rather than high‐level emotion categories. By integrating these multi‐level methods, this study aims to elucidate the neural dynamics of empathy processing across the ALT spectrum, offering refined empirical support for continuity models of autism.

## 2 Methods

### 2.1 Participants

An a priori power analysis was conducted using G*Power (Version 3.1.9.7) to determine the required sample size for a 2 × 2 mixed‐design analysis of variance (ANOVA). The analysis revealed that a total sample of 34 participants is needed to detect a medium‐sized interaction effect (*f* = 0.25) with 80% power at a significance level of *α* = 0.05. A total of 1273 questionnaires were administered to university student volunteers. Following the exclusion of 20 individuals who self‐reported a history of psychiatric disorders, the final screening sample comprised 1253 participants (543 identified males, 700 identified females). Participants were assessed using the AQ, and individuals falling within the top and bottom 10% of the AQ score distribution were identified. From these high‐ and low‐scoring subsets, 21 individuals were randomly selected for the high‐ALT group and 19 for the low‐ALT group, yielding a final sample of 40 participants who completed the study between November 2024 and January 2025.

All 40 participants were right‐handed, had normal or corrected‐to‐normal vision, and reported no history of neurological or psychiatric disorders. All participants provided written informed consent before the experiment began. The high‐ALT group comprised 21 participants (five identified males, 16 identified females; *M*_age_ = 19.67 ± 1.89) with AQ scores ranging from 130 to 152 (*M*_AQ_ = 137.86 ± 5.80). The low‐ALT group consisted of 19 individuals (seven identified males, 12 identified females; age: *M*_age_ = 19.68 ± 1.86) with AQ scores from 85 to 103 (*M*_AQ_ = 97.58 ± 5.92). All participants in the study self‐identified as Asian and were of Han ethnicity. The study was approved by the Institutional Review Board of East China Normal University (HR2‐0253‐2022).

### 2.2 Questionnaires

The autism spectrum quotient assesses ALT (L. Zhang et al. 2016). The questionnaire consists of 50 items across five subscales: social skills, attention switching, attention to detail, communication, and imagination. Participants rated each item on a four‐point Likert scale (1 = “definitely disagree” to 4 = “definitely agree”), yielding total scores from 50 to 200. Higher scores indicate a greater degree of ALT.

### 2.3 Apparatus and Stimuli

**FIGURE 1:**
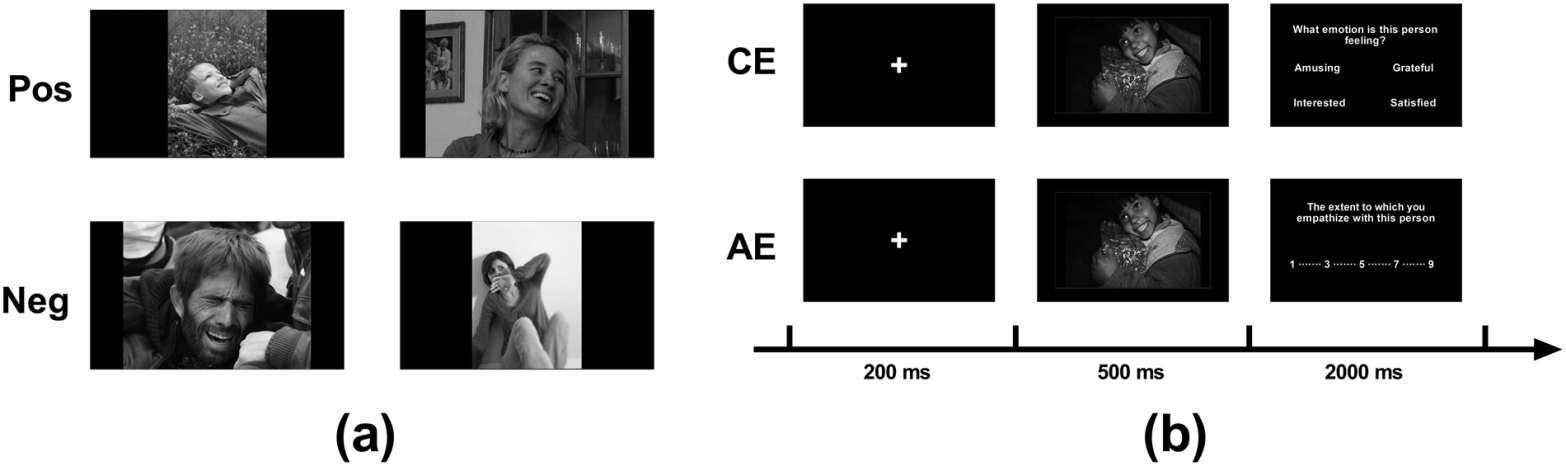
Experimental stimuli and task paradigm. (a) Examples of positive (top) and negative (bottom) valence stimuli used to elicit empathy. (b) Single‐trial procedure. In the CE task, participants identified the depicted emotion; in the AE task, they rated their own emotional response.

The experiment was programmed and presented using E‐Prime 2.0 on a computer. Participants sat at a distance of 45 cm from a 21.5‐in. Dell monitor (1024 × 768 resolution, 60 Hz refresh rate). Stimuli were images depicting individuals in various emotional scenarios. We selected an initial pool of 40 images based on a previous study (Foell et al. 2018; Pang et al. 2022). To ensure validity, four researchers reached a consensus to select the final 20 test images, confirming that each unambiguously conveyed either a positive or negative emotion. To enhance ecological validity, the images featured individuals of diverse genders, ages, and ethnicities. The final set comprised 10 images of positive emotional scenes and 10 of negative scenes. We used 20 additional, unique images for the practice phase. To control for the influence of color, we converted all images to grayscale JPEG format with a resolution of 650 × 300 pixels (Figure 1a).

After the experiment, participants validated the stimuli by rating the 20 test images for emotional valence and arousal on a seven‐point Likert scale (1 = “not at all” to 7 = “very intense”). The data from three participants (two from the high‐ALT group, one from the low‐ALT group) were excluded due to incomplete ratings. Mean (SD) valence ratings for positive images were 4.88 (0.88) for the high‐ALT group and 5.16 (0.78) for the low‐ALT group; for negative images, ratings were 5.18 (0.74) and 5.36 (0.93), respectively. Mean arousal ratings were similarly recorded.

### 2.4 Procedure

The experiment consisted of two blocks designed to measure CE and AE. Block order was counterbalanced across participants. Before each experimental block, participants completed a 20‐trial practice phase. The trial procedure is depicted in Figure 1b.

In the CE task, participants inferred the emotions of the individuals shown in the images. Each trial began with a 200‐ms central fixation cross (0.6° × 0.6°), followed by a 500‐ms presentation of an emotional scene image (17.1° × 10.3°). After the image disappeared, an array of four emotion words (one target, three distractors) appeared in the four quadrants of the screen. Participants then had 2000 ms to select the best‐matching word via a keypress (f, v, j, or n).

The AE task measured participants' emotional resonance with the individuals depicted in the images. The trial structure was identical to that of the CE task, except for the response stage. After the 500‐ms image presentation, a nine‐point Likert scale (1 = “not at all”; 9 = “very intense”) appeared on the screen for the response. Participants then rated the intensity of their emotional response within a 2000‐ms window.

Each block contained 320 trials (20 images × 16 repetitions), presented in a pseudorandomized order. The entire experiment lasted approximately 30 min. Participants were compensated for their time.

### 2.5 EEG Recording and Preprocessing

We recorded EEG data using a 64‐channel Ag/AgCl system (Brain Products, Gilching, Germany). Electrodes were arranged according to the international extended 10–20 system. The online reference was FCz, and the ground was AFz. To monitor eye movements, we recorded vertical electrooculography (VEOG) from an electrode placed below the right eye. We maintained electrode impedance below 10 kΩ and recorded data at a 500‐Hz sampling rate.

For the multivariate decoding analysis, the EEG data underwent a specific preprocessing pipeline. First, the continuous data were re‐referenced to the common average. Following this, the data were downsampled to 200 Hz, and a band‐pass filter of 0.5–20 Hz was applied. Artifact rejection was performed, resulting in the exclusion of less than 1% of the trials across the sample. Subsequently, Independent Component Analysis was run to identify and remove components associated with ocular and muscle artifacts. These steps were all implemented within the EEGLAB toolbox (Delorme and Makeig 2004), running on MATLAB 2022 (MathWorks, MA, USA), and were supplemented by custom scripts for batch processing. All trials not rejected during artifact correction were included in the final analysis to maximize statistical power.

### 2.6 Analysis

#### 2.6.1 Behavioral Data Analysis

We analyzed behavioral data to compare performance on the CE and AE tasks between the high‐ALT and low‐ALT groups. For the CE task, the dependent variables were mean reaction time (RT) and mean accuracy (ACC) for correct trials. For the AE task, the dependent variable was the mean empathy rating. We analyzed RT, ACC, and empathy ratings using separate 2 (Emotion Type: positive vs. negative) × 2 (Group: high‐ALT vs. low‐ALT) mixed‐design ANOVAs. We followed up significant interactions with simple effects analyses. We applied the Bonferroni correction to all post hoc multiple comparisons. We performed all statistical analyses in IBM SPSS Statistics 26.0.

#### 2.6.2 Multivariate Pattern Analysis

Using the MVPA‐light toolbox in MATLAB 2022 (Treder 2020), we trained a linear SVM classifier at each time point from −200 to 500 ms relative to stimulus onset. We implemented an eight‐fold cross‐validation scheme, wherein we iteratively trained the classifier on seven randomly partitioned data subsets and tested it on the remaining subset. To ensure robust results, we repeated this entire procedure eight times, each with a different random data partition. We quantified classifier performance using the area under the curve (AUC). An AUC value significantly above the chance level of 0.5 indicates that the neural pattern at that time point contains decodable emotion information.

Furthermore, we conducted a temporal generalization analysis to assess the stability of neural representations over time. In this analysis, a classifier trained at a specific time point (t_train) was used to decode data from all other time points (t_test). This procedure generates a temporal generalization matrix in which the diagonal represents the standard time–resolved decoding accuracy. Significant off‐diagonal decoding indicates a stable neural representation shared between the training time point (t_train) and the testing time point (t_test).

#### 2.6.3 Representational Similarity Analysis

To examine the information represented in neural activity, we conducted a time‐resolved RSA. This method assesses the correspondence between the geometry of neural representations and the geometry predicted by theoretical models, revealing what information is encoded at different time points.

**FIGURE 2:**
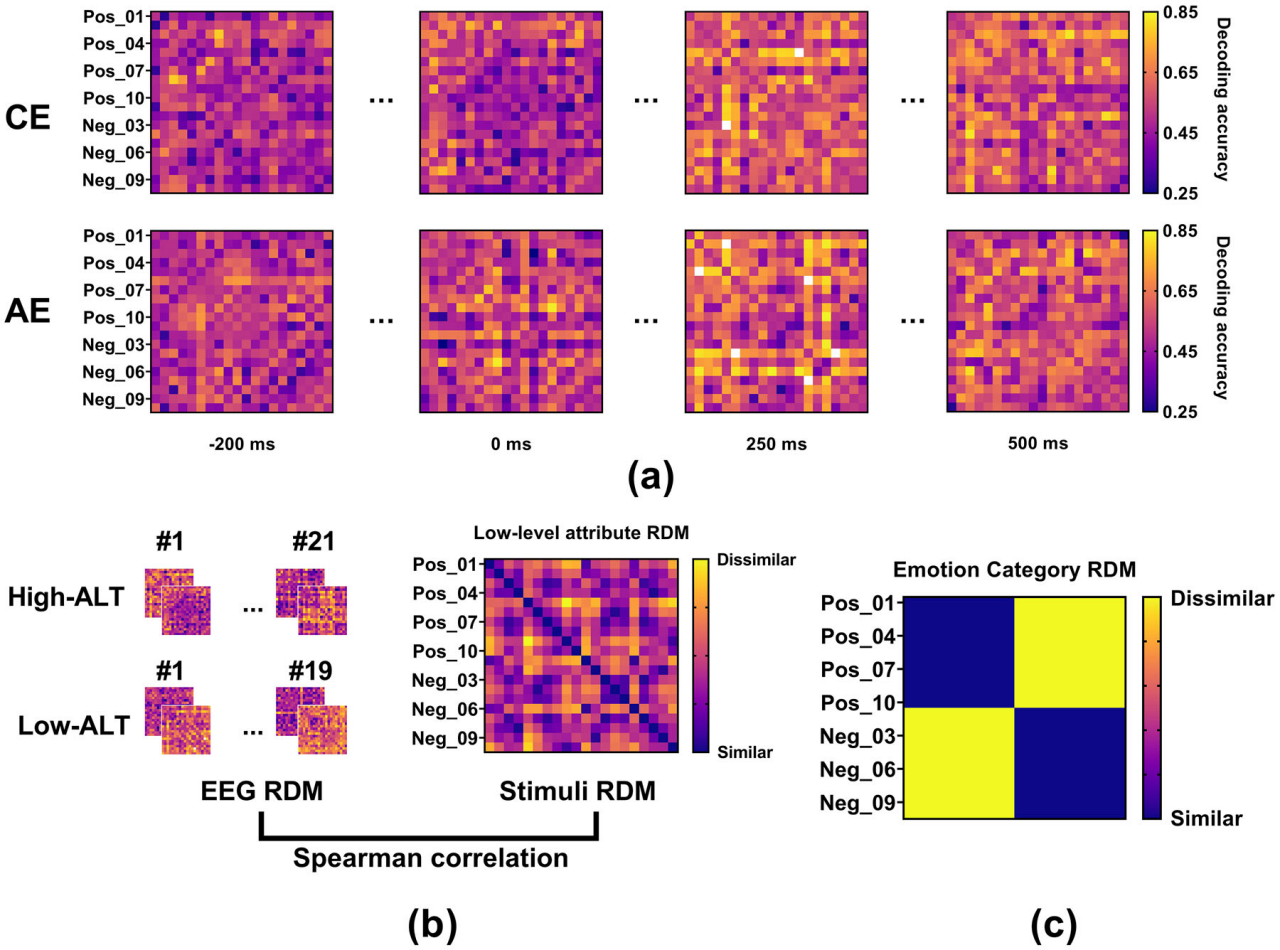
Representational similarity analysis (RSA) schematic. (a) Example of EEG RDMs from a single participant, where color indicates neural distinguishability between image pairs. (b) The RSA procedure, correlating time‐resolved neural RDMs with a low‐level attribute model RDM. (c) The emotion category model RDM is based on emotional valence.

First, we constructed a 20 × 20 neural representational dissimilarity matrix (RDM) for each participant at each time point. We defined neural dissimilarity between any pair of images as the decoding accuracy (ACC) of a linear discriminant analysis classifier trained to distinguish between their corresponding patterns of neural activity. Higher decoding accuracy indicated greater dissimilarity between the neural representations. We repeated this pairwise decoding for all 190 unique image pairs at each time point. The resulting ACC values populated the off‐diagonal cells of the 20 × 20 symmetric RDM. This process yielded a time series of neural RDMs for each participant, with each RDM capturing the representational geometry of the 20 stimuli at a specific time point (Figure 2a).

Second, we constructed two theoretical model RDMs to test hypotheses about the information encoded in the neural signal. The low‐level attribute model RDM was based on visual features. To create it, we calculated the Euclidean distance between all image pairs in a 2D feature space defined by mean luminance and contrast. This process resulted in a 20 × 20 symmetric RDM representing a model driven purely by low‐level physical properties (Figure 2b). The emotion category model RDM represented the hypothesis that the brain encodes emotional valence. In this model, the dissimilarity for image pairs within the same emotion category was 0.5, while the dissimilarity for pairs from different categories was 1. This model RDM thus partitioned stimuli into positive and negative clusters (Figure 2c).

Finally, we correlated the neural RDMs with the model RDMs at each time point. To do this, we vectorized the lower triangle of each neural RDM and model RDM, then computed Spearman's rank correlation between the resulting vectors at each time point. This analysis yielded a time course of correlation values representing the similarity between the neural data and each theoretical model. This time course revealed when the neural representational structure corresponded more closely to low‐level physical properties versus high‐level emotion categories.

#### 2.6.4 Statistical Testing

The statistical significance of MVPA and RSA time courses was assessed using a non‐parametric, cluster‐based permutation test (1000 permutations) implemented in MATLAB to correct for multiple comparisons across time. Clusters were formed by grouping contiguous time points that exceeded an initial statistical threshold (*p* < 0.05, one‐tailed), with test statistics computed at each time point using a Wilcoxon signed‐rank test. The sum of the statistical values within each cluster (i.e., the “maxsum” approach) served as the cluster‐level statistic. The final significance of each observed cluster was determined by comparing its statistic against the null distribution of maximum cluster statistics. For MVPA, this null distribution was generated by randomly flipping the signs of the trial labels to test against chance decoding accuracy (AUC = 0.5). For RSA, it was generated by randomly shuffling the rows and columns of the model RDM to test against a null hypothesis of zero correlation.

To capture individual differences in neural dynamics, we performed a single‐subject level analysis. For each participant, we identified time windows of significant decoding using the same cluster‐based permutation approach (*p* < 0.05). From these significant clusters, we extracted three metrics: Onset Latency (start time of the first significant cluster), Sustainability (total duration of all significant clusters), and Peak Latency (time point of maximum AUC). Independent samples *t*‐tests were used to compare these metrics between the groups. Finally, Spearman correlation analyses were conducted to examine the relationships between these neural metrics and behavioral indices (specifically, the difference scores between positive and negative conditions, i.e., positive—negative).

## 3 Results

### 3.1 Behavioral Results

**FIGURE 3:**
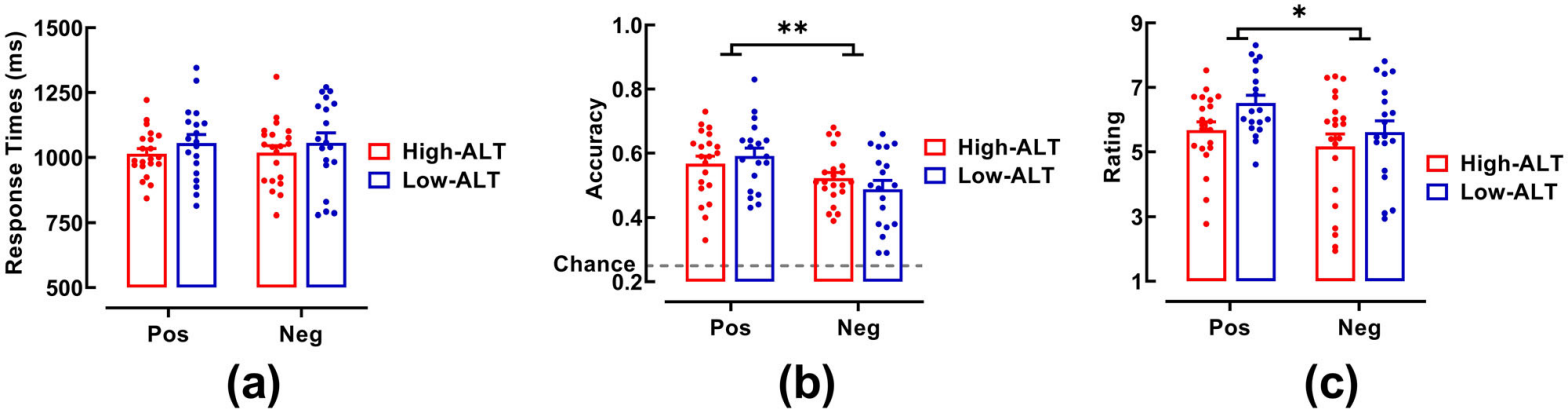
Behavioral performance by group. Bar charts with individual data points displaying the distributions of (a) RT and (b) ACC in the CE task, and (c) subjective empathy ratings in the AE task. Error bars represent the standard error of the mean. *Note*: ** *p* < .01, * *p* < .05.

The distributions of RT and ACC for the CE task, as well as subjective ratings for the AE task, are visualized in Figure 3.

#### 3.1.1 CE Task

As shown in Figure 3a, for RT, the ANOVA revealed no significant main effect of emotion type (*F*(1, 38) = 0.038, *p* = 0.847, *η_p_* ^2^ = 0.001) or group (*F*(1, 38) = 0.048, *p* = 0.828, *η_p_* ^2^ = 0.001), nor was there a significant interaction (*F*(1, 38) = 1.701, *p* = 0.200, *η_p_* ^2^ = 0.043). To further assess the evidence for this lack of group difference, we conducted a Bayesian analysis, which yielded a Bayes factor (*BF*_01_) of 2.688, providing anecdotal‐to‐moderate support for the null hypothesis.

As shown in Figure 3b, for ACC, a significant main effect of emotion type was observed (*F*(1, 38) = 11.176, *p* = 0.002, *η_p_* ^2^ = 0.227), with participants showing higher accuracy for positive scenes (0.58 ± 0.11) compared to negative scenes (0.51 ± 0.10). Crucially, neither the main effect of group (*F*(1, 38) = 0.955, *p* = 0.335, *η_p_* ^2^ = 0.025) nor the interaction (*F*(1, 38) = 0.032, *p* = 0.858, *η_p_* ^2^ = 0.001) reached significance. A supplementary Bayesian independent samples *t*‐test on the group effect yielded a *BF*_01_ of 4.153, providing moderate evidence in favor of the null hypothesis and substantiating the behavioral equivalence between groups.

#### 3.1.2 AE Task

As shown in Figure 3c, for subjective ratings, there was a significant main effect of emotion type (*F*(1, 38) = 7.313, *p* = 0.010, *η_p_* ^2^ = 0.161), indicating stronger empathic resonance for positive scenes (6.08 ± 1.18) than for negative scenes (5.38 ± 1.65). The main effect of group (*F*(1, 38) = 3.163, *p* = 0.083, *η_p_* ^2^ = 0.077) and the interaction (*F*(1, 38) = 0.572, *p* = 0.454, *η_p_* ^2^ = 0.015) were nonsignificant. A Bayesian analysis yielded a *BF*_01_ of 1.097, suggesting no conclusive evidence for a group difference.

### 3.2 MVPA Results

#### 3.2.1 Group‐Level Temporal Decoding

**FIGURE 4:**
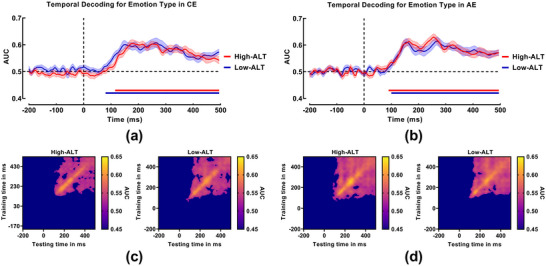
Neural dynamics of emotion category encoding. Time‐resolved decoding accuracy (AUC) for the high‐ALT (red) and low‐ALT (blue) groups during the (a) CE and (b) AE tasks. Horizontal solid lines indicate time windows of significant within‐group decoding (*p* < 0.05, cluster‐corrected). Shaded areas represent the standard error of the mean. Temporal generalization matrices for the (c) CE and (d) AE tasks. Areas enclosed by contours indicate decoding performance significantly above chance.

Time‐resolved decoding analyses examined the temporal dynamics of emotional information processing across the ALT spectrum. As shown in Figure 4a, in the CE task, the neural activity patterns in the low‐ALT group began to significantly differentiate between positive and negative emotions at 80 ms post‐stimulus (AUC > 0.5, *p* < 0.05, cluster‐corrected), with this discriminability persisting until 500 ms. In comparison, the onset of significant decoding in the high‐ALT group was observed numerically later, beginning at 115 ms. While this 35‐ms difference suggests a qualitative lag in the early neural encoding of emotion categories for high‐ALT individuals, it is important to note that direct statistical comparison between groups did not yield significant differences, likely due to high individual variability (see Section 3.2.3). In the AE task (Figure 4b), the two groups exhibited highly similar decoding profiles. The low‐ALT group showed significant differentiation starting at 90 ms, while the high‐ALT group began at 100 ms, indicating that the timing of early automatic affective processing is largely comparable between groups.

#### 3.2.2 Group‐Level Temporal Generalization

To assess the stability of neural representations over time, we conducted a temporal generalization analysis.

In the CE task (Figure 4c), the generalization patterns revealed subtle differences in the temporal extent of neural encoding. While both groups exhibited somewhat fragmented generalization clusters within the 100–500 ms window, a key distinction was observed in the onset of generalization. The cluster for the low‐ALT group extended earlier in time, with the leading edge (the “tip” of the generalization matrix) emerging closer to stimulus onset. In contrast, the significant generalization cluster for the high‐ALT group was confined to a later time window, lacking the early temporal extension observed in the low‐ALT group. This pattern aligns with the time‐resolved decoding results, further suggesting a delayed engagement of stable emotion representations in high‐ALT individuals.

In the AE task (Figure 4d), the temporal generalization matrices were remarkably similar and robust across groups. Both high‐ALT and low‐ALT individuals exhibited large, continuous, and intact clusters of significant generalization throughout the 100–500 ms range. This indicates that unlike in the CE task, the neural representation of emotion in the AE task is highly persistent, stable, and efficiently maintained regardless of ALT levels.

#### 3.2.3 Single‐Subject Temporal Decoding Metrics and Behavioral Correlations

To quantify neural dynamics at the individual level, we extracted three metrics (Onset Latency, Peak Latency, and Sustainability) for each participant who exhibited significant decoding performance.

In the CE task, the successful decoding of emotion valence was achieved in 57.14% of the high‐ALT group (12/21 participants) and 63.16% of the low‐ALT group (12/19 participants). Among these decoders, the high‐ALT group exhibited a numerically later onset (*M*_onset_ = 86.25 ± 57.96 ms) and shorter sustainability (*M*_sustain_ = 360.00 ± 77.31 ms) compared to the low‐ALT group (*M*_onset_ = 60.00 ± 58.66 ms; *M*_sustain_ = 390.00 ± 108.92 ms).

In the AE task, decoding success rates were higher, with 71.43% of the high‐ALT group (15/21) and 73.68% of the low‐ALT group (14/19) showing significant decoding. The temporal metrics were highly similar between the groups (high‐ALT: *M*_onset_ = 76.00 ± 33.07 ms, *M*_sustain_ = 406.67 ± 48.17 ms; Low‐ALT: *M*_onset_ = 86.43 ± 28.98 ms, *M*_sustain_ = 397.50 ± 42.91 ms).

Independent samples *t*‐tests revealed no significant group differences for any of these metrics (*p* > 0.05), potentially due to the reduced effective sample size after excluding non‐decoders and the high inter‐individual variability.

**FIGURE 5:**
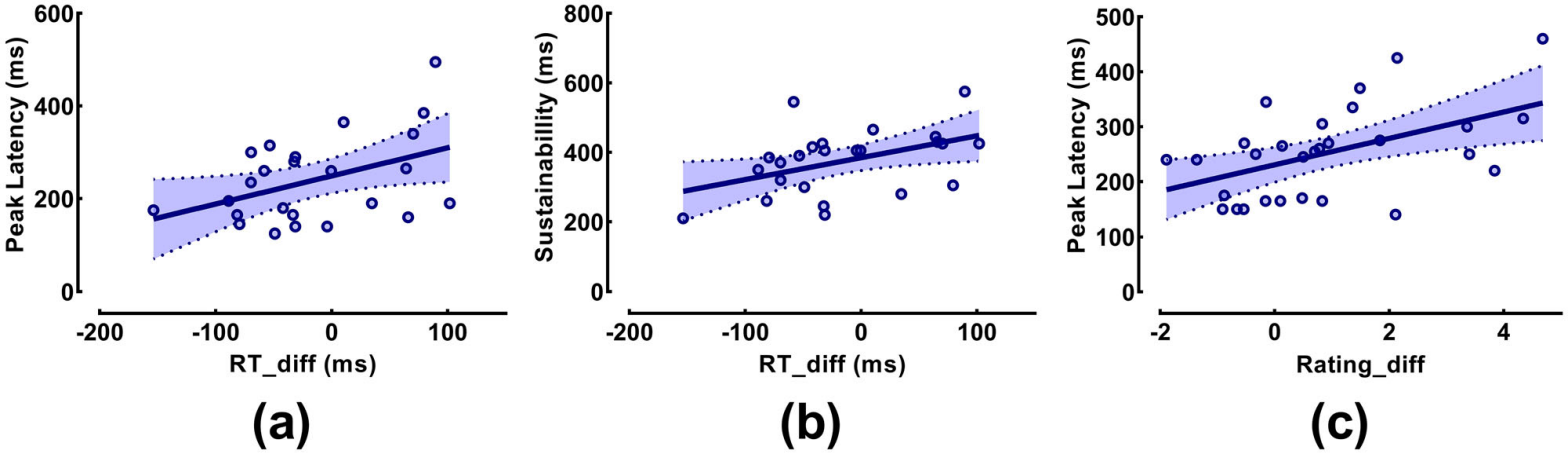
Correlations between neural metrics and behavioral performance. Scatter plots illustrate the linear relationships between single‐subject neural decoding parameters and behavioral indices. (a) In the CE task, Peak Latency significantly predicted RT differences (RT_diff; *R* ^2^ = 0.187, *p* = 0.035). (b) In the CE task, Sustainability was positively correlated with RT_diff (*R* ^2^ = 0.202, *p* = 0.028). (c) In the AE task, Peak Latency was associated with empathy rating differences (Rating_diff; *R* ^2^ = 0.247, *p* = 0.006). Each dot represents an individual participant with significant neural decoding. Solid lines indicate the best‐fit linear regression; shaded areas represent 95% confidence intervals. *Note*: RT_diff and Rating_diff refer to the difference in values between positive and negative conditions.

Crucially, as shown in Figure 5, these neural metrics significantly predicted behavioral performance. In the CE task, the difference in RT between positive and negative trials (RT_diff) was positively correlated with both Peak Latency (*r* = 0.433, *p* = 0.035; Figure 5a) and Sustainability (*r* = 0.449, *p* = 0.028; Figure 5b). This suggests that individuals with more sustained and later‐peaking neural representations showed a larger RT discrepancy between emotional conditions. In the AE task, a significant positive correlation was found between Peak Latency and the difference in empathy ratings (Rating_diff; *r* = 0.496, *p* = 0.006; Figure 5c), indicating that a later peak in neural processing is associated with a greater differentiation in reported empathy intensity.

### 3.3 RSA Results

To elucidate the informational content of neural representations, we correlated the neural RDM at each time point with two theoretical models: the low‐level attribute model and the emotion category model.

#### 3.3.1 Neural Encoding of Low‐Level Attributes

**FIGURE 6:**
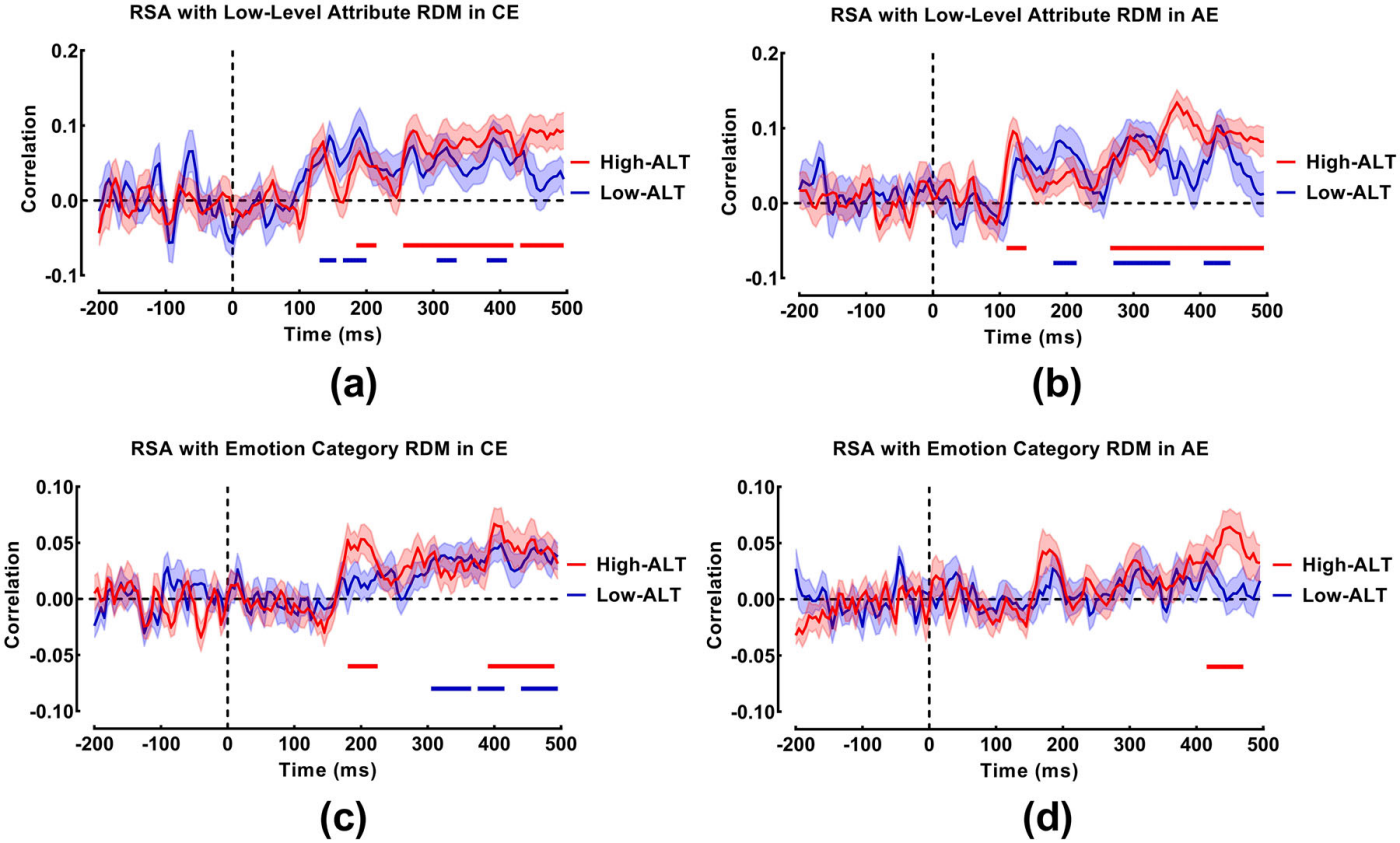
RSA results showing the correspondence between neural and model RDMs. The curves display the time‐resolved Spearman's correlation coefficients between the EEG RDM and the two theoretical model RDMs for the high‐ALT (red) and low‐ALT (blue) groups. Horizontal solid lines indicate time windows of a significant within‐group correlation (*p* < 0.05, cluster‐corrected). Shaded areas represent the standard error of the mean. The association with the low‐level attribute model is shown for the (a) CE and (b) AE tasks. The association with the emotion category model is shown for the (c) CE and (d) AE tasks.

In the CE task (Figure 6a), both groups showed sustained sensitivity to low‐level visual features, but with distinct temporal patterns. The high‐ALT group exhibited significant correlations across broad time windows (185–215 ms, 255–420 ms, and 430–495 ms), indicating that physical stimulus properties influenced their neural activity throughout nearly the entire processing epoch. The low‐ALT group also showed multiphasic encoding (130–155 ms, 165–200 ms, 305–335 ms, and 380–410 ms), but this processing notably ceased earlier (by 410 ms) compared to the high‐ALT group.

In the AE task (Figure 6b), the difference was more pronounced. The high‐ALT group exhibited a remarkably sustained neural response to low‐level attributes, particularly in a late, continuous time window extending from 265 to 495 ms (in addition to an early 110–140 ms cluster). In contrast, the low‐ALT group showed a more fragmented pattern (180–215 ms, 270–335 ms, 380–410 ms, 405–445 ms). This suggests that in the AE task, the neural activity of high‐ALT individuals remains heavily driven by superficial physical properties of the stimuli well into the late cognitive evaluation stage.

#### 3.3.2 Neural Encoding of Emotion Categories

In the CE task (Figure 6c), the encoding of emotion categories revealed divergent strategies. Surprisingly, the high‐ALT group showed an early cluster of significant representation (180–225 ms), followed by a long latency period before re‐emerging at a later stage (390–490 ms). The low‐ALT group, conversely, exhibited significant encoding primarily in the later stages (305–365 ms, 375–415 ms, 440–495 ms). Although the onset appeared earlier in the high‐ALT group, the substantial temporal gap (approximately 165 ms) between their early and late clusters suggests a less continuous integration of emotional information compared to the temporally clustered processing observed in the low‐ALT group.

In the AE task (Figure 6d), the correspondence with the emotion category model was markedly weaker than in the CE task for both groups. The high‐ALT group exhibited only a single, late‐stage significant cluster (415–470 ms), while the low‐ALT group showed no clusters surviving the cluster‐based correction. This general lack of robust categorical encoding, despite significant valence decoding in MVPA, suggests that the neural computations underlying affective resonance may differ fundamentally from those supporting explicit emotion identification. Specifically, the representational geometry during AE may not align strictly with the binary “positive vs. negative” categorical structure, a possibility we explore further in Section 4.

## 4 Discussion

This study investigated the neural dynamics of CE and AE in individuals with high and low ALT. Our primary finding revealed a dissociation: while the high‐ and low‐ALT groups showed comparable behavioral performance of empathy, their underlying neural processing patterns were significantly different. This pattern of “behavioral equivalence, neural heterogeneity” suggests that high‐ALT individuals employ compensatory neural strategies to achieve performance comparable to their low‐ALT counterparts (de Jonge et al. 2025). These results support the conclusion that ALT influences neural processing efficiency and highlight how behavioral measures alone can mask significant latent differences in social cognition (Oliver et al. 2016).

### 4.1 Heterogeneity in CE Processing

Our finding of equivalent behavioral performance on the CE task aligns with previous studies showing no performance deficits in high‐ALT or ASD populations within certain experimental paradigms (McKenzie et al. 2022; Oliver et al. 2016). However, our neurophysiological data expose the significant processing costs underlying this behavioral equivalence.

First, regarding sensory processing, our analysis revealed that the high‐ALT group's neural activity was predominantly driven by the stimuli's low‐level physical attributes (RSA results). This reliance on superficial features was corroborated by our univariate findings, where the high‐ALT group failed to show the early valence‐dependent modulation of the N100 component observed in the low‐ALT group (Supporting Information). These converging results suggest that during the initial stages of CE, high‐ALT individuals' attention is inefficiently allocated, being captured by low‐level sensory features at the expense of salient emotional cues (Y.‐T. Fan et al. 2014).

Consequently, the formation of emotion category representations was markedly disrupted. While RSA indicated a transient early encoding of emotion categories in the high‐ALT group, this representation was notably discontinuous, followed by a substantial latency gap before re‐emerging at a later stage. Consistent with this, our MVPA results showed numerically later onset times and less stable temporal generalization patterns. This fragmented “early transient, late re‐engagement” pattern is likely a hallmark of a cognitive compensation process. It refines the traditional view of “impaired CE” (aan het Rot and Hogenelst 2014; Brazil et al. 2024; Fletcher‐Watson and Bird 2020) by suggesting the deficit manifests as reduced processing efficiency and the use of circuitous strategies, rather than an absolute inability to perform the task. This observation aligns with ERP studies linking empathy deficits to alterations in late components (e.g., LPP, P3) associated with conscious evaluation (Y.‐T. Fan et al. 2014; W. L. Li et al. 2023; X. Li et al. 2020). From a neural network perspective, these processing‐pattern differences may stem from atypical functioning of prefrontal cortical regions (e.g., the mPFC) responsible for executive control (de Waal and Preston 2017; Y. Fan et al. 2011).

### 4.2 Heterogeneity in AE Processing

In the AE task, the lack of group differences in behavior and early neural processing is consistent with studies reporting intact AE in individuals with ASD or high‐ALT (Pouw et al. 2013; Shirayama et al. 2022; Ziermans et al. 2019). Specifically, both the comparable N100/N250 amplitudes (Supporting Information) and the similar MVPA decoding onsets suggest that the initial and automatic affective resonance, a process supported by subcortical structures such as the amygdala and insula (de Waal and Preston 2017; Decety and Michalska 2010; Dvash and Shamay‐Tsoory 2014), is not significantly modulated by the degree of autistic traits.

However, our RSA results provided deeper insight into the qualitative nature of AE processing. Notably, unlike the CE task, the AE task elicited minimal to no significant encoding of explicit emotion categories in either group. This null finding is theoretically revealing: it suggests that in the AE task, which involves monitoring one's own continuous emotional resonance rather than categorizing an external stimulus, the brain may not rely on rigid categorical representations.

Crucially, despite this shared lack of categorical coding, the high‐ALT group exhibited a distinct anomaly in sensory processing. While the low‐ALT group efficiently disengaged from the stimuli's low‐level physical attributes, the neural activity in the high‐ALT group remained driven by these superficial features for a much longer duration (extending late into the processing window). This prolonged focus on sensory details, coupled with the absence of stable high‐level integration, may mechanistically explain the unique empathic difficulties reported in this population. The “bottom‐up” sensory overload might interfere with subsequent cognitive regulation, potentially leading to personal distress or emotional flooding rather than adaptive, other‐oriented empathic concern (Shalev et al. 2022; Smith 2009; Ziermans et al. 2019).

### 4.3 Theoretical Implications

Behaviorally, this study found no significant differences between the high‐ and low‐ALT groups on CE and AE tasks yet revealed marked divergences in their underlying neural processing. This pattern of “behavioral equivalence, neural heterogeneity” invites interpretation through several competing theoretical lenses, including the ToM deficit model, the double empathy problem, and the empathy imbalance hypothesis. While our findings could be framed as a “bidirectional mismatch” (Milton 2012; W. Zhang et al. 2023) or as an imbalance between CE and AE (Rueda et al. 2015; Shalev and Uzefovsky 2020), we propose an alternative interpretation that refines the ToM deficit model. We argue that the observed neural differences do not reflect a simple impairment in the final CE outcome but rather a fundamental divergence in the cognitive mechanisms employed to achieve it. Building on research that localizes the CE deficits of high‐ALT individuals to later processing stages (W. L. Li et al. 2023; X. Li et al. 2020), our findings specify that this phenomenon manifests as a compensatory reliance on more prolonged and resource‐intensive cognitive evaluation to construct an accurate emotional representation. In contrast, the low‐ALT group appears to rely on more rapid, automatic processing (supported by the early N100 modulation observed in our supplementary analysis). Conversely, the processing pathways for AE appear more congruent between the groups, suggesting the primary divergence lies within the cognitive domain (Dziobek et al. 2008; Grove et al. 2014). Therefore, our findings do not refute a ToM‐related difficulty but instead recharacterize it: the “deficit” may lie not in the final capacity for understanding but in the automaticity and efficiency of the underlying cognitive processes (Baron‐Cohen and Wheelwright 2004; Rogers et al. 2007). This perspective shifts the focus from whether high‐ALT individuals can understand others' mental states to how they achieve this understanding, offering a more precise target for future research and intervention (Brazil et al. 2024; Fletcher‐Watson and Bird 2020).

### 4.4 Empathy and Individual Traits

Although this study interprets neural heterogeneity in the context of ALT, it is likely modulated by confounding factors. Alexithymia, a trait characterized by difficulty identifying and describing one's own emotions, is a prominent co‐occurring factor (Bird and Cook 2013). Indeed, previous research has consistently demonstrated a high degree of comorbidity between alexithymia and elevated autistic traits (B.‐L. Le et al. 2024; Ziermans et al. 2019). This connection is critical because the “alexithymia hypothesis” posits that many empathy deficits attributed to autism, particularly in CE, may instead stem from difficulty processing one's own feelings (Bird and Cook 2013; Mazza et al. 2014). This mechanism is plausible given the substantial overlap between the neural substrates of interoception (e.g., the insula and anterior cingulate cortex) and empathy (Singer et al. 2004).

However, this relationship is complex, as some research indicates that ALT continues to predict empathy deficits even after controlling for alexithymia (Vilas et al. 2021; Ziermans et al. 2019). Beyond alexithymia, callous‐unemotional (CU) traits are also strongly associated with deficits in AE (Jones et al. 2010). Crucially, this negative association is markedly amplified in individuals with high ALT, suggesting a synergistic detrimental effect on emotional resonance (Pasalich et al. 2014). These findings suggest that empathy is affected by distinct, interacting traits: ALT may primarily drive deficits in CE, CU traits may underpin impairments in AE, and alexithymia may act as a powerful mediator, particularly for CE (Brazil et al. 2024; Brett and Maybery 2022; Fletcher‐Watson and Bird 2020; Jones et al. 2010). Therefore, future research must systematically disentangle the unique and interactive effects of these constructs.

### 4.5 Constraints on Generality

The present findings should be considered in light of several key limitations. First, our conceptualization of empathy is a key limitation. We mapped CE to emotion recognition and AE to emotional contagion. It is debatable whether this operationalization measures pure empathy or a more generalized social‐emotional processing dysfunction (Georgiou et al. 2019; W. Zhang et al. 2023). Second, specific methodological choices constrained our findings. The use of static images limits the task's ecological validity (Schnitzler and Fuchs 2024). Furthermore, the multifaceted empathy test is susceptible to social desirability bias (Oliver et al. 2016; Schnitzler and Fuchs 2024). Additionally, the great difficulty of the emotion recognition task resulted in relatively low behavioral accuracy across participants. While performance remained well above chance level, the restricted variance in accuracy scores likely limited our ability to detect significant correlations between ACC and neural metrics. Finally, the rapid task pace required for EEG may have masked subtle behavioral differences.

Several limitations concerning our sample composition warrant caution when interpreting the results. Primarily, our reliance on a non‐clinical university student sample restricts the direct generalizability of our findings to individuals with a clinical diagnosis of ASD. Our use of an extreme group design, while effective for contrasting distinct ALT phenotypes, resulted in a bimodal distribution of AQ scores. This precluded whole‐sample correlation analyses across the full ALT spectrum and might have restricted the detection of linear trait–behavior relationships.

Moreover, significant neural decoding clusters were identified in approximately 50%–60% of the participants in each group. This led to a reduced effective sample size for the single‐subject metric extractions and brain–behavior correlation analyses, potentially limiting the statistical power to identify more subtle quantitative differences between the groups. Furthermore, our sample was predominantly female, with a limited number of male participants. This gender imbalance is a notable limitation, as a substantial body of research has documented significant gender differences in empathy (Shalev and Uzefovsky 2020). Consequently, our findings may not fully represent the patterns present in male participants, potentially overlooking gender‐specific nuances in the neural mechanisms of empathy.

Critically, the high co‐occurrence of alexithymia in individuals with high ALT our high‐ALT group presents a significant confound. This overlap makes it difficult to attribute the observed neural patterns solely to ALT. Consequently, future research must disentangle these constructs using larger, targeted samples and advanced statistical models (Lassalle et al. 2019; Nicholson et al. 2018). To advance the field, studies should employ more naturalistic paradigms, such as virtual reality, to improve ecological validity (Donaldson et al. 2022). Future studies should build a more comprehensive model of empathy by integrating multi‐level data, combining objective neurophysiological recordings with subjective assessments from both self‐ and other‐reports (Fletcher‐Watson and Bird 2020; Pasalich et al. 2014).

## Author Contributions

L.Z. and Y.J. made equal contributions to this manuscript. L.Z. contributed to formal analysis, methodology, and writing – original draft. Y.J. contributed to conceptualization and data curation. Z.C. contributed to data curation, formal analysis, methodology, and writing – review and editing. Y.S. contributed to conceptualization, funding acquisition, investigation, resources, supervision, and writing – review and editing.

## Funding

This research was part of the Fundamental Research Funds for the Centrsal Universities, Southwest University, China, Grant/Award Number: SWU2209326, 2022, and the Fundamental Research Funds for the Central Universities, Shanghai Lideshuren Humanities and Social Sciences Research Base Program, Grant/Award number: 2021/2022.

## Ethics Statement

The study was approved by the Institutional Review Board of East China Normal University (HR2‐0253‐2022).

## Conflicts of Interest

The authors declare no conflicts of interest.

## Supporting information




**Supplementary Material**: brb371236‐sup‐0001‐SuppMat.docx

## Data Availability

The stimuli and behavioral data in this study are publicly available on the Open Science Framework at https://osf.io/m7g8j/. The code generated for this study is available on request to the corresponding author.
