# Supplementary material for: Neural Heterogeneity Underlying Behavioral Equivalence: A Dynamic Neuro‐Decoding Study of Cognitive and Affective Empathy in Relation to Autism‐Like Traits
Source: Brain Behav. 2026 Jan 28;16(2):e71236. doi: 10.1002/brb3.71236 (PMC12848529; doi:10.1002/brb3.71236)
Supplement: Supplementary file 1 — Supplementary Material: brb371236‐sup‐0001‐SuppMat.docx [file BRB3-16-e71236-s001.docx]

**Supplemental Materials**

# Overview

This section provides the detailed results of the traditional univariate Event-Related Potential (ERP) analyses, serving as a complement to the multivariate findings (MVPA and RSA) presented in the main text. To investigate the specific temporal dynamics of empathy processing across the Autism-like Traits (ALT) spectrum, we focused on two key frontal-parietal components: the N100 and the N250. The selection of these components is grounded in a theoretical framework distinguishing between automatic emotional resonance and cognitive evaluation.

First, we analyzed the N100 component to index the early, automatic stage of empathy. Previous research suggests that the N100 reflects the initial "emotional contagion" or "emotional sharing" experience, a bottom-up process driven by early attentional allocation to emotional cues rather than conscious cognitive appraisal (Almeida et al., 2024; Groen et al., 2013). Specifically, frontal-parietal N100 responses are sensitive to emotional valence and facilitate early pain perception and emotional sharing (Meng et al., 2019). Examining the N100 allows us to test the hypothesis that early automatic resonance remains intact in high-ALT individuals, consistent with findings of negligible group differences in early ERP components (Li et al., 2020).

Second, we investigated the N250 component to evaluate the transition toward cognitive processing. While the N250 time window indicates early emotional arousal (Fan et al., 2014), it is also strongly associated with the cognitive evaluation aspects of empathy, such as mentalizing (Groen et al., 2013). Empirical evidence indicates that N250 amplitude positively correlates with empathy scores (Almeida et al., 2024), making it a critical marker for the "cognitive empathy system" (Groen et al., 2013). By comparing these specific components between high- and low-ALT groups, we aim to validate whether the "behavioral equivalence" observed in our study is supported by preserved early neural mechanisms (N100) or compensated by distinct patterns during the cognitive evaluation stage (N250).

# Methods

## Participants

The participants included in this supplementary analysis are identical to those described in the main text. The sample consisted of 40 university students, divided into a high-ALT group (*n* = 21) and a low-ALT group (*n* = 19) based on their AQ scores. All participants met the inclusion criteria detailed in the main manuscript.

## EEG Recording and Preprocessing

EEG data acquisition followed the same protocol described in the main text (64-channel Brain Products system, 500 Hz sampling rate). Preprocessing for the univariate ERP analysis was conducted in MATLAB using the EEGLAB toolbox with parameters optimized for ERP extraction. The continuous data were re-referenced to the common average and filtered with a 1–30 Hz band-pass filter. Data were segmented into epochs from -200 to 500 ms relative to stimulus onset, followed by a baseline correction using the -200 to 0 ms interval. Artifacts were removed via Independent Component Analysis (ICA) and visual inspection, resulting in a mean trial rejection rate of 5.2%. Only trials with correct responses (for the CE task) or completed ratings (for the AE task) were retained for analysis.

## ERP Analysis

To investigate the temporal dynamics of empathy at the physiological level, we analyzed specific ERP components identified in the Overview. Based on visual inspection of the grand-average waveforms and previous literature (Almeida et al., 2024; Fan et al., 2014; Groen et al., 2013), a central electrode cluster (Cz, C1, C2) was selected for analysis. The N100 component was defined as the mean amplitude within the 110–140 ms time window, indexing early emotional resonance and attentional allocation. The N250 component was defined within the 230–270 ms window, reflecting the cognitive evaluation of emotion categories. For each task (CE and AE) and each component, mean amplitudes were submitted to a 2 (Emotion Type: positive vs. negative) × 2 (Group: high-ALT vs. low-ALT) mixed-design ANOVA.

# ERP Results

## CE Task

The analysis of the N100 component revealed a significant main effect of emotion type, *F*(1, 38) = 4.970, *p* = .032, *η_p_*^²^ = .116, and a significant interaction between emotion type and group, *F*(1, 38) = 5.820, *p* = .021, *η_p_*^²^ = .133. The main effect of group was not significant, *F*(1, 38) = 1.690, *p* = .201, *η_p_*^²^ = .043. Simple effects analysis revealed distinct processing patterns between groups: the low-ALT group exhibited significantly more negative N100 amplitudes for positive scenes (-3.43 ± 1.91 *μ*V) compared to negative scenes (-2.89 ± 1.82 *μ*V; *p* = .003). In contrast, the high-ALT group showed no significant differentiation between positive (-2.40 ± 1.91 *μ*V) and negative scenes (-2.42 ± 1.81 *μ*V; *p* = .895), suggesting a reduced sensitivity to emotional valence at this early perceptual stage (Figure S1a).

For the N250 component, there was a significant main effect of emotion type, *F*(1, 38) = 39.566, *p* < .001, *η_p_*^²^ = .510, indicating that positive scenes elicited more negative amplitudes (-3.69 ± 2.57 *μ*V) than negative scenes (-2.74 ± 2.08 *μ*V) across all participants. Neither the main effect of group, *F*(1, 38) = 0.141, *p* = .709, *η_p_*^²^ = .004, nor the interaction, *F*(1, 38) = 0.330, *p* = .569, *η_p_*^²^ = .009, was significant (Figure S1a).

## AE Task

The analysis of the N100 component revealed no significant main effects of emotion type, *F*(1, 38) = 1.417, *p* = .241, *η_p_*^²^ = .036, group, *F*(1, 38) = 3.173, *p* = .083, *η_p_*^²^ = .077, or their interaction, *F*(1, 38) = 0.080, *p* = .778, *η_p_*^²^ = .002 (Figure S1b).

For the N250 component, there was a significant main effect of emotion type, *F*(1, 38) = 106.770, *p* < .001, *η_p_*^²^ = .738. Similar to the CE task, positive scenes elicited significantly more negative amplitudes (-3.51 ± 2.33 *μ*V) than negative scenes (-2.61 ± 2.04 *μ*V). The main effect of group, *F*(1, 38) = 0.960, *p* = .334, *η_p_*^²^ = .025, and the interaction, *F*(1, 38) = 1.305, *p* = .260, *η_p_*^²^ = .033, were not significant (Figure S1b).


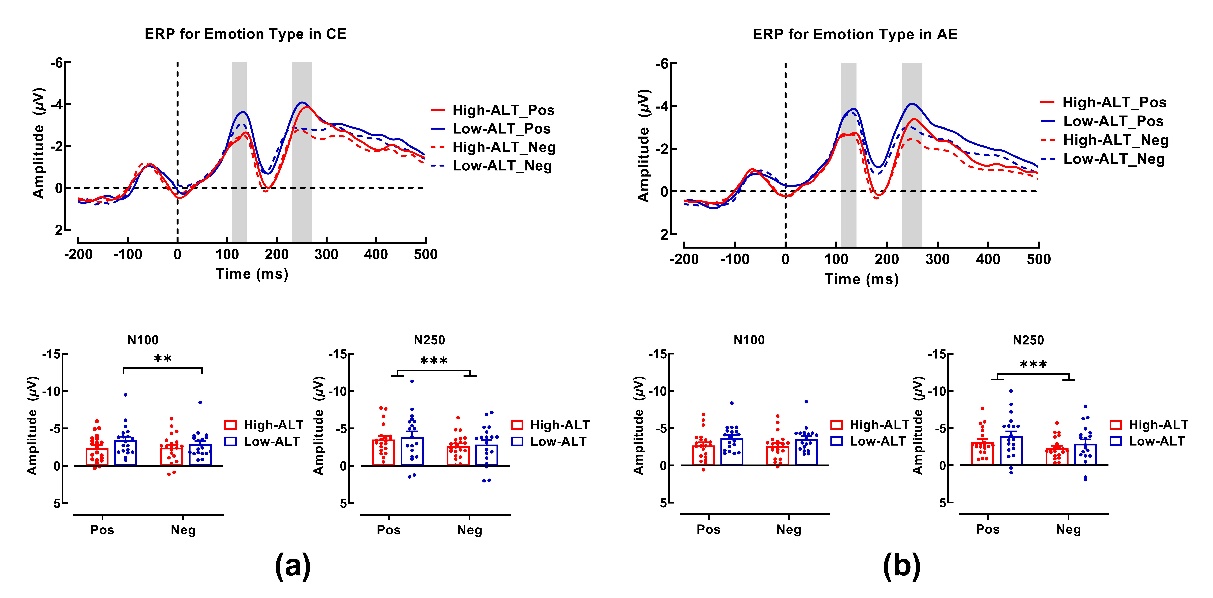


Figure S1. Grand-average ERP waveforms and mean amplitudes. (a) Waveforms elicited at the central electrode cluster (Cz, C1, C2) during the CE task. Solid lines represent positive stimuli and dashed lines represent negative stimuli for the High-ALT (red) and Low-ALT (blue) groups. Gray shaded regions indicate the time windows for the N100 (110–140 ms) and N250 (230–270 ms) components. Bar charts show the mean amplitudes for each component across groups and emotion types. (b) Corresponding ERP waveforms and mean amplitudes during the AE task. Error bars represent the standard error of the mean. Significance levels: ****p* < .001, ***p* < .01.

# Supplementary Discussion

The ERP findings provide complementary univariate evidence for the "behavioral equivalence, neural heterogeneity" pattern observed in our multivariate analyses. In the CE task, the significant Group × Emotion type interaction on the N100 component reveals a divergence in early perceptual processing. While low-ALT individuals showed rapid discrimination of emotional valence as early as 100 ms, high-ALT individuals failed to exhibit this early modulation. This absence of N100 differentiation aligns with our RSA findings, which indicated that the high-ALT group's early neural activity was driven predominantly by low-level physical attributes rather than emotion categories. This convergence suggests that the high-ALT group exhibits an initial deficit in allocating attention to salient emotional cues (Almeida et al., 2024; Li et al., 2020). However, the subsequent N250 component showed intact valence differentiation in both groups. Given that the N250 indexes cognitive evaluation and mentalizing (Groen et al., 2013), this "absent N100, intact N250" pattern strongly supports a compensatory mechanism: high-ALT individuals may bypass early automatic emotional tagging, relying instead on later-stage cognitive resources to achieve accurate emotion recognition.

In the AE task, the absence of group differences in both N100 and N250 amplitudes corroborates the view that the core neural machinery for affective resonance remains largely intact in high-ALT individuals (aan het Rot & Hogenelst, 2014; Li et al., 2020; Pouw et al., 2013; Shirayama et al., 2022; Ziermans et al., 2019). The N100 results suggest that the bottom-up "emotional contagion" mechanism functions typically, allowing for immediate emotional sharing regardless of autistic traits. Similarly, the comparable N250 responses indicate that the magnitude of neural engagement during the cognitive evaluation of one's own emotional response is preserved. However, it is important to note that while these univariate ERP amplitudes (reflecting the magnitude of response) were equivalent, our main text's multivariate analyses (reflecting the pattern stability and information content) revealed fragmentation in the high-ALT group. Together, these multi-level data suggest that while the "quantity" of neural activation (ERP) in AE is sufficient, the "quality" or stability of the underlying representation (MVPA/RSA) may be less efficient in high-ALT individuals.

# Reference

aan het Rot, M., & Hogenelst, K. (2014). The Influence of Affective Empathy and Autism Spectrum Traits on Empathic Accuracy [Article]. *Plos One*, *9*(6), Article e98436. <https://doi.org/10.1371/journal.pone.0098436>

Almeida, R., Prata, C., Pereira, M. R., Barbosa, F., & Ferreira-Santos, F. (2024). Neuronal Correlates of Empathy: A Systematic Review of Event-Related Potentials Studies in Perceptual Tasks. *Brain Sciences*, *14*(5), Article 504. <https://doi.org/10.3390/brainsci14050504>

Fan, Y. T., Chen, C. Y., Chen, S. C., Decety, J., & Cheng, Y. W. (2014). Empathic arousal and social understanding in individuals with autism: evidence from fMRI and ERP measurements. *Social Cognitive and Affective Neuroscience*, *9*(8), 1203-1213. <https://doi.org/10.1093/scan/nst101>

Groen, Y., Wijers, A. A., Tucha, O., & Althaus, M. (2013). Are there sex differences in ERPs related to processing empathy-evoking pictures? *Neuropsychologia*, *51*(1), 142-155. <https://doi.org/10.1016/j.neuropsychologia.2012.11.012>

Li, X., Li, Z. S., Xiang, B. Y., & Meng, J. (2020). Empathy for pain in Individuals with autistic traits influenced by attention cues: Evidence from an ERP study. *Acta Psychologica Sinica*, *52*(3), 294-306. <https://doi.org/10.3724/sp.J.1041.2020.00294>

Meng, J., Shen, L., Li, Z., & Peng, W. (2019). Top-down effects on empathy for pain in adults with autistic traits. *Scientific Reports*, *9*(1), 1–13. <https://doi.org/10.1038/s41598-019-44400-2>

Pouw, L. B. C., Rieffe, C., Oosterveld, P., Huskens, B., & Stockmann, L. (2013). Reactive/proactive aggression and affective/cognitive empathy in children with ASD. *Research in Developmental Disabilities*, *34*(4), 1256-1266. <https://doi.org/10.1016/j.ridd.2012.12.022>

Shirayama, Y., Matsumoto, K., Hamatani, S., Muneoka, K., Okada, A., & Sato, K. (2022). Associations among autistic traits, cognitive and affective empathy, and personality traits in adults with autism spectrum disorder and no intellectual disability [Article]. *Scientific Reports*, *12*(1), Article 3125. <https://doi.org/10.1038/s41598-022-07101-x>

Ziermans, T., de Bruijn, Y., Dijkhuis, R., Staal, W., & Swaab, H. (2019). Impairments in cognitive empathy and alexithymia occur independently of executive functioning in college students with autism. *Autism*, *23*(6), 1519-1530. <https://doi.org/10.1177/1362361318817716>
